# Devil Is in the Details: Use of Wild Food Plants in Historical Võromaa and Setomaa, Present-Day Estonia

**DOI:** 10.3390/foods9050570

**Published:** 2020-05-04

**Authors:** Raivo Kalle, Renata Sõukand, Andrea Pieroni

**Affiliations:** 1 University of Gastronomic Sciences, Piazza Vittorio Emanuele 9, 12042 Pollenzo, Italy; a.pieroni@unisg.it; 2 DAIS-Department of Environmental Sciences, Informatics and Statistics, Ca’ Foscari University of Venice, Via Torino 155, 30172 Mestre, Italy

**Keywords:** wild food plants, Estonia, diachronic analysis, local ecological knowledge, influence of literature, borders, ethnic groups, ethnobotany

## Abstract

Biodiversity needs to be preserved to ensure food security. Border zones create high but vulnerable biocultural diversity. Through reviewing scattered historical data and documenting the current use of wild food plants among people currently living in historical Setomaa and Võromaa parishes, we aimed to identify cross-cultural differences and diachronic changes as well as the role borders have played on the local use of wild plants. The Seto have still preserved their distinctive features either by consciously opposing others or by maintaining more historical plant uses. People historically living in Setomaa and Võromaa parishes have already associated the eating of wild plants with famine food in the early 20th century, yet it was stressed more now by the Seto than by Estonians. Loss of Pechory as the center of attraction in the region when the border was closed in the early 1990s brought about a decline in the exchange of knowledge as well as commercial activities around wild food plants. National support for businesses in the area today and the popularity of a healthy lifestyle have introduced new wild food plant applications and are helping to preserve local plant-specific uses in the area.

## 1. Introduction

The regional importance of the use of wild food plants has been particularly studied in recent years in the context of the Mediterranean diet [1,2] and the references therein. Following the example of the Mediterranean diet, enthusiastic Nordic chefs and promoters of food culture created the trans-national concept “New Nordic Cuisine” in 2004. The manifesto of this “New Nordic Cuisine” consists of 10 points that emphasize local, clean, healthy, traditional, wild, and high-quality food uses in modern gastronomy [3,4]. However, this concept has failed to fulfil its “Nordic” purpose, as national and state interests in the region are of greater importance [5]. In Estonia, the national campaign “Estonian Food” is a very strong identity marker, as are several regional campaigns, among them “Uma Mekk” in Võru County. Within this framework, the use of wild food plants in top restaurants has started to spread in Estonia [6], as they seek to stand out amid intense competition.

Biodiversity needs to be preserved to ensure food security [7]. Due to the need to evaluate ecosystem services, biodiversity has been seen as a potential source of wild food plants in the context of Eastern Europe [8]. Within Estonia, the most biodiverse areas are semi-natural grasslands [9]. Forest management, however, may decrease the availability of ecosystem goods in areas where humans collect them [10]. A study by Hadjichambis et al. [11] reveals that the existence of favorable conditions is not yet a prerequisite for the use of wild food species but rather is strongly linked to the traditions, environment, and cultural heritage of each specific region. Therefore, it is not enough to preserve the agro-biological diversity of food; cultural specificities also need to be preserved [11]. Therefore, when the habit of picking wild plants from nature disappears, the great potential offered by biodiversity is not utilized [12]. Thus, both biological and cultural diversity are important for food security, which should be studied as a complex, i.e., within the framework of biocultural diversity [13].

Many studies have emphasized the importance of comparative analysis of plant use for understanding the biocultural adaptations of different linguistic groups and in different historical periods within Mediterranean countries [1,14,15]. Yet, in the Baltic States and Nordic countries, the use of wild food plants has been little studied. Even in a small country like Estonia, bioculture is highly region dependent: Compare, for example, species-rich marine west Estonia and less species-rich continental east Estonia. In addition, the areas bordering Russia have been greatly influenced by Russian culture, while western Estonia and especially the islands in the Baltic Sea have been influenced by Scandinavian culture. While studies on the use of wild food plants in western Estonia, especially on Saaremaa Island, have already been published [16,17,18], they do not reflect the situation in other parts of the country. Moreover, there is very little information on the eating of wild plants in southeast Estonia in descriptive historical ethnographic books [19,20,21]. Current uses are briefly addressed only in ethnic cookbooks written for both the local community and tourists [22,23,24].

Culturally, the most distinct ethnic minority in Estonia are the Seto (here and hereafter we use the dialectal name; the official name in Estonian is Setu). As the Seto live in a border area between three countries (Latvia, Estonia, and Russia), the issue of border identity has been actively studied there since the beginning of the 21st century. Assmuth [25,26] found that in the Soviet era, when there was no border, the economic situation was better on the Estonian side compared with the Russian side. As a result, border residents travelled frequently from Russia to Estonia, the so-called near abroad, to buy food and consumer goods. Since the border was closed, there has been a surge in Estonia’s economic prosperity, whereas on the Russian side, it has tended to remain the same or become more disadvantaged (e.g., limited choice of goods in shops). Assmuth [25,26] also found that the desire for the return of freedom of movement is stronger among the Seto people living in Russia, as the environment there does not support their culture.

South-eastern Estonia, especially Setomaa, has been actively studied by researchers for over 100 years. Setomaa was *Terra Incognita* until the beginning of the 20th century, when folklorist Jakob Hurt (1839–1907), with his articles and books, highlighted the cultural peculiarities of the people living there. Although he was the first to record the use of wild plants by the Seto, Hurt and all subsequent researchers were keener to record folk songs and tales, and not the practical side of the interaction between people and their environment. Hurt’s opinion was that the Seto were at least a few hundred years “behind” Estonians in their development at that time. This view was still valid among scientists until the late 1930s. The reason why the Seto, who have been on the frontier of Western and Eastern cultures for centuries, were studied was because they were considered “aboriginal Estonians” whose study would contribute to a better understanding of Estonian (not Seto) history and culture [27].

Through reviewing scattered historical data and documenting the current use of wild food plants among people currently living in historical Setomaa (here and hereafter we use the dialectal name) and neighboring historical Võromaa (here and hereafter we use the dialectal name) parishes, we aim to address the following research questions:

1. What kinds of differences, if any, in the use of wild food plants currently exist between the two ethnolinguistic groups which were considered very different 100 years ago?
2. What kinds of changes and the causes of those changes can be identified when comparing current results with historical wild food plant use in the region?
3. If and how a recently imposed border and being cut-off from the regional attraction center affect the use of wild food plants?
4. What role, if any, do wild food plants play in the current local gastronomy of the region?

## 2. Materials and Methods

### 2.1. The Region

#### 2.1.1. Nature

The nature of Võromaa and Setomaa has been shaped by the Haanja Upland (the highest point is 318 m above sea level, which marks the highest elevation in the Baltic States) and Lake Peipus (consisting of Lake Peipsi, Lämmi, and Pihkva, which together cover a total area of 3555 km^2^), one of the largest inland water bodies in Europe. Historically, the area has been dominated by coniferous forests, with low-yielding sandy soils, pine forests, and fertile areas with spruce forests. Setomaa is also characterized by an abundance of water-meadows, which were grazed in the past but are now losing their species richness as many economic activities have ended [28]. There are large cranberry-rich bogs in Võromaa, especially by Lake Peipsi. The vegetation of south-eastern Estonia is one of the oldest in the country as it was the first region that began to be depleted of ice about 14,000 years ago, while the last freeing of Estonian areas occurred about 10,000 years ago. Today, however, it is one of the least species-rich areas in Estonia. In the 20th century, rapid changes in the environment have led to the disappearance of native plants. For example, in the Estonian part of Setomaa, 717 native species (including subspecies) had been registered by 1971, of which 149 species had disappeared and an additional 88 species had become very rare by 2005 [29]. The disappearance of species has continued in the 21st century, during which time common species have also begun to decline in number [30].

#### 2.1.2. Population and Administrative Division

**Figure 1:**
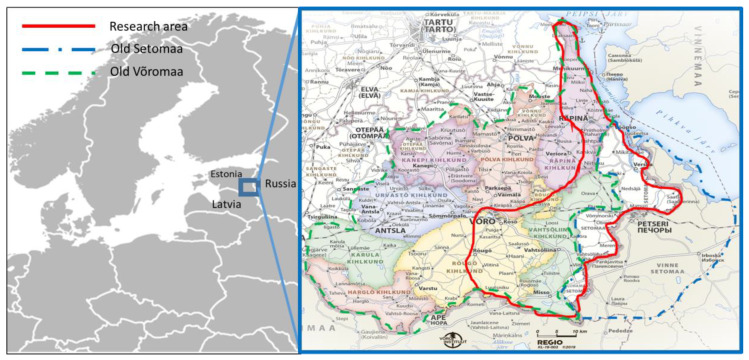
Research area: the approximate area is encircled by a red line. On the right are the administrative boundaries of historical Võromaa and Setomaa, in which place names are written in the Võro dialect (Source: Võro Instituut).

The research area extends across two historical areas: Võromaa and Setomaa (Figure 1). Võromaa represents the area in which the Võro dialect is predominantly spoken. It largely coincides with the historical Livonian Governorate region of Kreis Werro. There are also many people who speak dialects other than Võro in the area, and therefore we will henceforth use the term Estonians to refer to people from this region. Setomaa is an area historically inhabited by speakers of the Seto language and Russian speakers. Today, it is considered an area that was established after the Estonian War of Independence in 1920 and existed until the end of World War II, when most of Setomaa was incorporated into the Russian SFSR on the order of Stalin. While the greatest part of historical Setomaa is today part of the Russian Federation, our fieldwork was carried out in the Estonian side of Setomaa.

Numerous administrative reforms have repeatedly changed the historical boundaries of the municipality as well as the county (Figure 1). According to the latest administrative reform in 2017, there are four official rural municipalities in the study area: Võru rural municipality (10,738 inhabitants), Setomaa rural municipality (3280 inhabitants), Rõuge rural municipality (5427 inhabitants), and Võru city (11,859). In Estonia, all ethnography and folkloristic research is based on the administrative divisions of the late 19th century. According to the administrative arrangements of that time, there were four parishes in our area of interest: Räpina, Vastseliina, Rõuge, and Põlva, the latter of which belonged to Kreis Werro.

Unlike Estonians, until the 1920s, the areas inhabited by Setos did not follow the legal norms of the Estonian or Livonian governorates but that of the Pskov Governorate of Pskov Province. The Seto lived mostly on lands belonging to Pechory Monastery. Although today the Seto consider themselves indigenous, the European Union does not officially consider them as such. In the Russian Federation, the Seto are considered a minority group whose culture and language are at risk.

In the territory of historical Võromaa, village and town schools were established in the 19th century and school subjects were taught in Estonian. The Seto then living in Pskov Province did not have schools that taught in their native language, and only a few Seto people could receive a formal education. As a result, by the beginning of the 20th century, the Seto were predominantly illiterate. Administratively belonging to Pskov Province brought about Russification; however, after the Estonian War of Independence (1920), when the historical settlement of the Seto was incorporated into the Republic of Estonia, the new government started to assign Seto surnames and establish village schools that taught in Estonian, at which time rapid Estonianization began [27,31,32]. Both Võro and Seto are commonly regarded as dialects of the Estonian language; however, the language carriers themselves consider them separate languages, and according to UNESCO, both are endangered languages. The Seto faith is a mixture of paganism and Christian Orthodoxy, whereas in neighboring Võromaa, Estonians are Lutheran and the Russians Orthodox Christians.

Throughout history, Setomaa and part of Võromaa have had a lower standard of living compared with the rest of Estonia. The main reasons for this are the low fertility of the soils and very small farmlands of these regions. Since farms were small, they have always been engaged in growing vegetables: Mainly cucumbers, onions, and strawberries [33,34,35]. No large-scale industrial production has existed in the area and the small industries, even today, are mostly involved in the production of small-scale crafts, especially ceramics. Today, forestry and the timber industry also provide jobs. Historically, trading and brokerage have also been an important source of income. Overpopulation and poverty have led to many waves of mass emigration. The first was from the end of the 19th century to the beginning of the 20th century, when during the Czarist Siberian colonization campaign, the Seto moved into the area between Yenisei Province (today Krasnoyarsk Krai), and the Kan and Mana rivers. Newcomers were exempt from state taxes, land was given free of charge and settlement infrastructure was supported [36]. Since the 1920s, labor migration has been actively practiced, mainly in the spring-summer-autumn period. Above all, the Seto travelled to the inlands of Estonia as well as to Latvia. In addition, the Seto relocated in order to work in the large industrial enterprises of northern Estonia, forming small but vivid communities in the nearby regions [37,38,39,40]. Migration has also occurred within Setomaa. When the majority of the area was incorporated into the Russian SFSR after the Second World War, administration came under Russian control. This and the closure of Estonian-language schools led to the first major movement of people to the Estonian side of the region. The second major migration to Estonia took place in 1997, when the border of the newly independent Republic of Estonia was effectively closed, and the Estonian state began to provide support for relocation [25]. The economic disadvantages of the area have persisted to the present day, which has led to the decline of younger people in the area due to migration to cities [31].

### 2.2. Fieldwork

Fieldwork was carried out in the summer of 2018 and 2019 and covered historical Võromaa and the portion of historical Setomaa, which is still part of present-day Estonia. The sample included people who had either lived their entire lives in the same area or had been studying or working elsewhere in Estonia and had now returned. The youngest interviewee was 35 years old, while the oldest was 93 years old; detailed demographical data for the interviewees is provided in Table 1.

We asked the interview questions in Estonian but urged interviewees to answer in their native dialect if this was more comfortable for them. The study goals were explained to the interviewees and oral or written consent was obtained. On permission of the participants, the interviews were recorded. The study was approved by the Ethics Committee of Università Ca’ Foscari and strictly followed the ethical guidelines outlined by the International Society of Ethnobiology [41]. We conducted semi-structured interviews lasting from 30 min to over 2 h covering food, medicinal, and other uses of plants.

For the wild food plant section of the interview, which was conducted first, initial “free listing” was followed by questions regarding specific foods or additives to specific foods that were made (soups, salads, fermentations, hot dishes, spices, etc.) and the wild food plants involved in their preparation. If some of the plants included in the free listing were not subsequently mentioned, the respondent’s attention was guided to those as well. We also asked where the person had learned the use and when exactly was the first/last use of the plant. The initial interview was followed, whenever possible (in ca 50% of cases), by a walk in the interviewee’s garden, surrounding meadows, or forest. Quite often people pointed out new plants or uses not mentioned during the main interview when they saw the plant in its natural habitat. We also carried out participant observation in local fairs, markets, and public festivals, and visited small producers, museums, and eateries in the area.

Plants were identified on site and, whenever possible, herbarium specimens and, if offered by interviewees, dry plant samples were collected. All voucher specimens and dried samples were stored at the Herbarium of Ca’ Foscari Univeristy of Vence (UVV) bearing herbarium numbers SE001–SE141 and SEDR001–SEDR051. If the specimen was not available, the plant was identified on the basis of its popular name and full description of the plant and its habitat. Latin plant names are provided according to The Plant List [42], and families are assigned according to the Angiosperm Phylogeny Website [43]. Some taxa were identified only on the genus/family level or given as a collective name as they are perceived this way by people, even if voucher specimens were present for specific species of the genus, in order to avoid over-identification.

### 2.3. Data Processing and Analysis

The field recordings were transcribed into text and in the few cases where recording was not allowed, the interview context was reconstructed on the basis of field notes as soon as possible following the interview. The data on the wild food plants was subsequently entered into an Excel spreadsheet based on detailed use records (DUR, a number of use records considering all details of use, e.g., the plant part and specific preparation involved sensu [16]). As wild food plants, we considered all plants used for food that grow without direct human involvement, including those naturalized or those cultivated for non-food proposes (e.g., [44]).

To understand if differences still exist between the groups on an ethno-linguistic level, we divided our interviewees into two groups (see Table 1) and analyzed the results on the following temporal scale:

Past uses (no longer in active use):

CH–abandoned in childhood and used by the informant’s parents, grandparents, or themselves as a child (circa 1940s–1950s),

AB–abandoned in adulthood (circa 1960s–1990s), and

AD–temporarily used in adulthood (circa 1970s–2000s).

Current uses:

AT–always used (used more or less continually from childhood to the present), and

NW–learned only recently (since circa 2000, but mainly the last 5 years).

We further compared past and current uses mentioned during interviews to qualitatively assess if and how a recently imposed border and being cut-off from the regional attraction center have affected the use of wild food plants within the lifetime of the person. In the comparative analysis, we used RAWGraphs [45].

### 2.4. Comparison with Historical Data

In the ethnographic literature, as well as in archival texts, we searched for data on Räpina, Vastseliina, Rõuge, and Põlva parishes and also Setomaa. We used articles [46,47,48,49,50,51] by Aliise Moora (1900–1996), a researcher of Estonian food culture. These articles were based on data collected before the Second World War by the Estonian National Museum (ERM) and the Estonian Folklore Archives (ERA). Before the war, and after, the collection of cultural heritage took place for the preservation of knowledge and the emphasis was mainly on quantity; in other words, to collect a lot of everything. The staff of the Archives published a new survey questionnaire each year and they were mostly engaged in archiving incoming information and preparing new survey plans but not their analysis. Moora later acknowledged the shortcomings of this earlier method of collecting food culture. There was no communication with the correspondents and the archive staff did not notice any limitations in the material submitted or the specifics that could have given the material a qualitative dimension (e.g., [52]). Thus, there were also major shortcomings in the collection of data on the consumption of wild species. Many lists were submitted by respondents, but the information on what the food was made of, how the plants were collected, or whether the listed plants were actually eaten and when was very scarce [47]. Therefore, the ethnographic material allows generalizations only in restricted cases, for example, regarding the use of the culturally most important wild berries [53].

In addition to the ethnographic literature, we also looked through the manuscript collections of the Estonian Folklore Archives (ERA) and the Estonian National Museum (ERM). The most important early ethnobotanical collection [54] was compiled by Gustav Vilbaste (1885–1967), a teacher, conservationist and botanist. Vilbaste began collecting popular plant names and plant uses as early as 1907 and he collected them until his death. He conducted fieldwork and also had a large network of correspondents with whom he actively communicated. From the earlier collections, we also looked through the aforementioned Jakob Hurt’s Folklore Collection [55] and the Estonian Folklore Archives Collection [56]. In the ERM, we reviewed data from the archive of correspondent responses up to the 1960s, mainly on the use of tea and herbs, to which Moora did not pay much attention [57]. Because the ethnobotanical material was collected using various methods and somewhat chaotically, it can only be used for qualitative analysis, while quantitative comparisons with the current data cannot be made. When analyzing popular plant names, we proceeded from the book: “Nomina vernacula plantarum Estoniae” [58].

## 3. Results and Discussion

**Figure 2:**
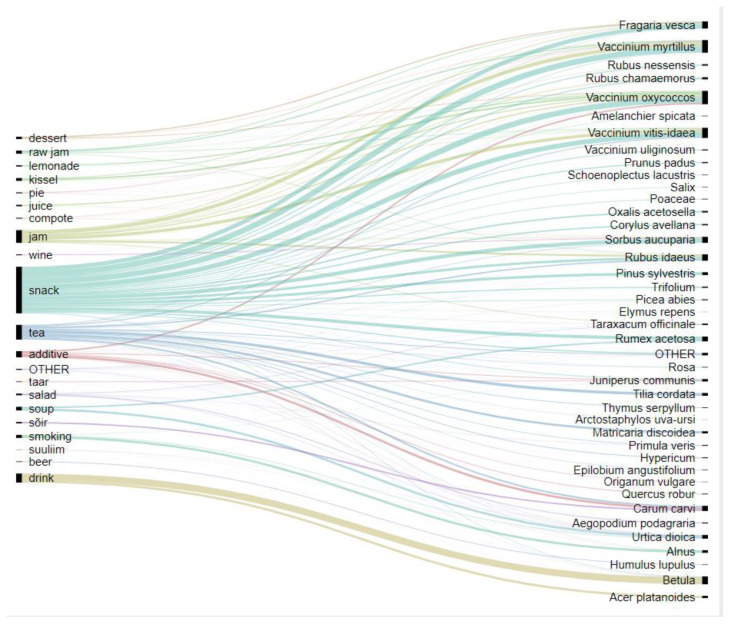
Alluvial diagram of the relationship between the food made and the wild taxa used by both Seto and Estonians together. “Other” refers to taxa/uses with less than 4 detailed use records (DUR).

Of the 63 taxa used in the region, 58 were identified on the species level, 4 on the genus level, and one on the family level (Table 2). Of the 26 families represented, the most numerous were Rosaceae (13 taxa), Lamiaceae and Ericaceae (6 taxa each), and Betulaceae and Poaceae (4 taxa each). The most commonly used taxa were three *Vaccinium* species, including *Vaccinium oxycoccos* (187 DUR), *Vaccinium myrtillus* (177 DUR), and *Vaccinium vitis-idaea* (142 DUR), followed by *Betula* (107 DUR) and *Fragaria vesca* (100). Therefore, the most widely used families were Ericaceae (526 DUR), Rosaceae (365 DUR), and Betulaceae (162 DUR). The most popular food categories were snacks (655 DUR), recreational tea (207 DUR), jam (178 DUR), and drinks (127 DUR) (Figure 2).

**Figure 3:**
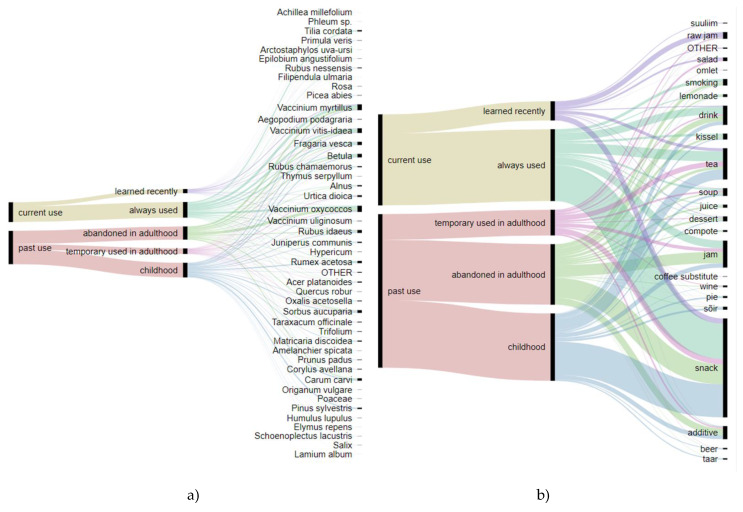
Alluvial diagrams outlining (**a**) taxa used and (**b**) food made depending on the time of use.

The use of plants has changed considerably over time: A smaller number of uses were reported as used now (37%, 603 DUR) compared to those used in the past (67%, 1017 DUR). The currently used taxa consist of a little less than 8% (127 DUR) that were learned recently and 30% (476 DUR) that were used throughout life. Past uses consist of those used only throughout childhood (28%, 446 DUR), those abandoned in adulthood (25%, 401 DUR), and those used only for short time, e.g., learned during adulthood but no longer used (10%, 170 DUR). The division of taxa and uses on a temporal scale shows that the abandonment or acquisition of uses takes place on a personal not community, level. The exceptions are a few taxa (*Salix* sp., *Schoenoplectus lacustris*, *Lamium alba*–all used as snacks) abandoned in childhood and the use of *Achillea millefolium* as recreational tea recently acquired by at least three people (Figure 3a, Table 2). On the level of use, the use of beer and beer-like drinks has been abandoned, while a new food, a cold soup called “suuliim”, has recently emerged and the freezing of (mainly) forest and bog berries has become popular (Figure 3b, Table 2).

Only a few forest berries (e.g., *Vaccinium vitis-idaea, Vaccinium myrtillus*) and bogs berries (e.g., *Rubus chamaemorus, Vaccinium oxycoccos*), as well as *Corylus avellana* seeds, were widely collected beyond “walking distance” or outside of random trips in the wild. Table 2 also lists the taxa that people have eaten elsewhere in Estonia if they lived outside of their current area for some time. These uses are exogenous and sporadic, such as eating *Rubus caesius* berries, drinking tea made from *Mentha aquatica*, and eating cooked *Elymus repens* roots. These plants are no longer eaten after returning to a childhood village. For the same reason, *Empetrum nigrum* has been eaten only by those who have lived near bogs, as their parents taught them to recognize the berries. In general, people knew and used more taxa that lived close to their home, and those living close to a bog ate more bog taxa than those who only went to pick specific berries seasonally.

The use of plants by children is a special category, in which uses were passed on only to other children and were not used in later life. Thus, the interviewees who spoke about the use of *Schoenoplectus lacustris, Equisetum arvense, Angelica sylvestris*, and some other childhood snacks had a difficult time recalling their names and were often unsure if the recalled name was correct, as such plants were little spoken about during adulthood. The use of certain plants was copied from other children, older than themselves, without even remembering the clear plant description at that moment. Age-related taste perception was also repeatedly emphasized, as interviewees stated that sour things are no longer desired, and that is why the eating of *Sorbus aucuparia* berries in particular has decreased. They are now being eaten frozen once the bitterness is gone. For the same reason, *Oxalis acetosella* is no longer eaten in adulthood. The same has been observed elsewhere in Estonia, such as on Saaremaa Island [16].

Industrial production has reduced the need for home-made winter conserves and fermented drinks. As in Saaremaa [16], home-made sauerkraut has virtually disappeared and been replaced by shop products. This is the reason why *Vaccinium oxycoccos* and *Carum carvi*, as additives to sauerkraut, were mainly used in the past. Additionally, jam rich in sugar is no longer made, not only because of the availability of jam in shops but also because of the health movement: Jams have been substituted by frozen berries. Our interviewees noted that in the past, fermented beverages, either beer or beer-like low-alcoholic beverages (*taar*), were very common, but today both beverages have been replaced by shop-bought drinks. For this reason, the previously abundant use of wild species as flavoring and preservatives has disappeared. While older people still remembered making fermented *taar*, no one remembered how to make the juniper drink historically typical of the region. The actual making of flavored fermented cereal drinks (*taar*, beer) has disappeared as such drinks are no longer made in homes.

Alcohol culture changed during the Soviet era: We were told that in the pre-Soviet period, vodka drinking was not widespread in villages. On the one hand, this was the result of an active abstinence movement and, on the other, the popularity of home-brewed beer. After World War II, alcohol began to be widely consumed. This custom became more popular after the mass migration of Slavic migrant workers to Estonia, when more people started to drink vodka. Therefore, during Soviet times, illegal alcohol called “handsa” (moonshine) was distilled at home. We were told that after the Moscow Olympics in 1980, when for a short time the shops sold imported berry-liqueurs, various previously unknown home-made berry-liquors emerged.

The planting of a woody tree species near a house has a great impact on its use. *Tilia cordata* is virtually absent in modern (managed) forests and its flowers are harvested from trees planted in gardens and ornamental plots. Planting trees on farms began in the 1930s with the so-called national home decoration campaign. In Soviet times, farm landscapes no longer focused on farmhouses, and new artificial attraction centers, so-called collective farm centers, were created. Lime trees were planted by schools and houses of culture, and their flowers are now being picked from trees in both large courtyards and parks. The same was true for the use of sap from *Acer platanoides*, which rarely grows large enough in modern forests, and thus the sap is taken from trees planted near the house. For a number of interviewees, the tree did not grow close to the house and no one brought it from far away, and as a result many had not even tried the sap.

*Carum carvi* has historically been one of the most widely used seasoning plants. However, there has been a drastic reduction, with only 10 DUR still used nowadays compared to the nearly 60 DUR reported for the past. Even among today’s users, only a few pick it from the wild regularly, while most often the seeds are bought from a pharmacy or shop. As in Saaremaa [16], the reason for the decline is that the species is disappearing from the wild mainly because of intensive mowing of roadsides and ceasing maintenance of natural meadows.

In historical Võromaa, smoking meat in a smoke sauna has been one of the most distinctive and well-known ways of preserving meat. Even today, this custom is one of the region’s trademarks [59]. Free-standing smoke ovens were first introduced to the region about 50 years ago. The archival data do not contain much information on meat smoking, but this was due to the specifics of the surveys.

### 3.1. Cross-Cultural Analysis

**Figure 4:**
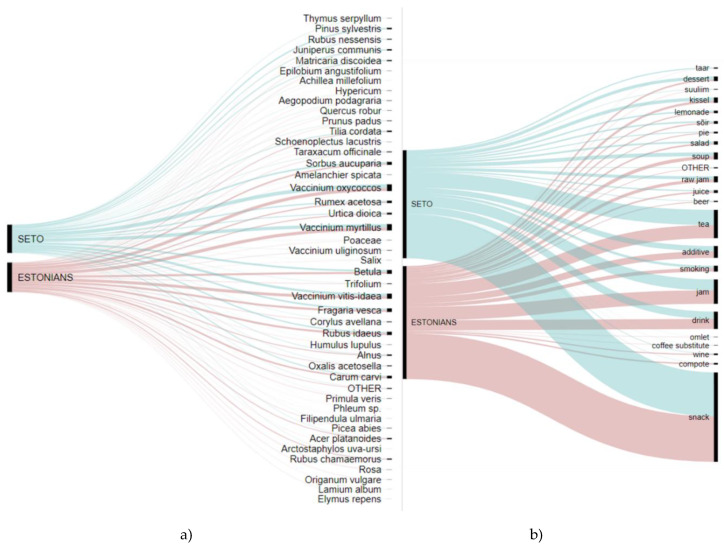
Alluvial diagrams representing the differences between the two groups for (**a**) taxa used and (**b**) food made.

Differences between the Seto and Estonians can be observed on the level of specific taxa or use-group of the plants (Figure 4), i.e., in the details, which need a qualitative approach and further explanation. There were differences on the taxa level depending on the availability of the taxa in the region. A perfect example is *Thymus serpyllum,* which grows in sandy soils in open areas, as this species is common in Setomaa but is only found in some places in Võromaa. Historically, this plant has had many popular names in the area; however, during fieldwork, only the Seto told us about the plant’s popular name, collection in nature, and use. Several people pointed out that *T. serpyllum* is disappearing from its usual habitat. Estonians grow this plant in the garden today, but they only use the name derived in the literature and they do not go out to pick it from the wild. Conversely, *Origanum vulgare* is common in meadows, roadside borders, and sparse forests. The use of *O. vulgare* as a tea and herb (for blood sausage) is quite common among Estonians, but we did not record such uses among the Seto. Today, the Seto grow this plant in the garden, using only the official name, but they have never picked it from the wild nor used it as a seasoning in blood sausage. The archives reveal that the first time blood sausages were made in Võromaa (Räpina parish) was in the late 19th century. They were undoubtedly made for Christmas and New Year’s; less was done on family holidays. In the 1940s in Võromaa, “vorstirohi” (*Origanum majorana*) was already being grown in herb gardens. However, the Seto that lived near Pechory began to put “vorstirohi” (probably also *O. majorana*) in blood sausages in the 1930s, yet it was bought in shops as a ready-made dry seasoning [57]. This may also be the reason why they did not substitute this seasoning later for the wild one (*O. vulgare*): They could not associate the use of shop-bought seasoning with the local plant.

Another example is that of *Rubus nessensis*, which is frequently found in south-eastern Estonia. However, this plant had the highest number of local plant names today among the Seto, who claimed to actively collect and eat its berries, while Estonians mentioned eating them solely when living in families together with the Seto. During Soviet times, a warning was circulated that all red berries were poisonous and as parents advised their children not to eat unknown berries, they were not picked. This may also explain why *Rubus saxatilis*, which is not now well known to Estonians and the Seto, and *Rubus nessensis*, which is not common among Estonians, are no longer eaten: The unknown berries were considered to be toxic.

The avoidance of red berries was, in fact, literature driven. While the earliest book introducing fruit use suggested that children should eat the fruits of *Maianthemum bifolium* [60], during the Soviet era, a widespread berry book [61] warned against eating potentially poisonous berries. This is probably the reason why such use is only recorded in the historical data. The same historical book stated that *Viburnum opulus* is poisonous and even birds do not want these berries [60], although for Russians it is quite common as a food plant [62]. The belief that the loss of interest in those berries is related to the reduction in Russian influence in the region is widespread, as historically, in Estonia, this berry has been eaten only in areas adjacent to Russia. Meanwhile, the Russian plant name is still in use: “it is *kalina*, *krasnaja kalina* [in Russian калина]. But we were always warned that they should not be touched, these are poisonous berries” (Seto man, born 1958).

Earlier Estonian literature has clearly had less influence on the Seto. The only herbal book published during the Soviet era, “Eesti NSV ravimtaimed”, recommended eating wild plants as a good source of vitamin C [63]; for example, *Primula officinalis* leaves had not been eaten before, but now they are used. The Seto, however, have not embraced this new use. Today’s ethnic food cookbooks point out that mashed potatoes with *P. officinalis* leaves are a local Midsummer’s Day food [23]. Other iconic examples are desserts (such as syrup) and recreational tea made from the flowers of *Filipendula ulmaria*, which has become a fashionable plant all across Estonia. The use of its leaves in green soups is also common nowadays in Võromaa [22]. The Seto interviewees knew it was popular but had not used it themselves. Only one interviewee recalled that her mother used this plant spontaneously (made tea) because it had flowers that smelled good. However, *Epilobium angustifolium*, which has become popular today due to Russian-language literature, was now used by both Estonians and the Seto (see also [64]). Additionally, the use of *Taraxacum officinale* was not reported in archival texts, and so its use is the result of later literature (ca 1980s). However, respondents noted that due to the bitter taste of this plant, it was only tried for a short time and is therefore not widely used today.

A taste-dependent difference, historically and still today, is that the Seto do not use the cones of *Humulus lupulus* in fermented beverages as they “cause headache”. There are many reports of the Seto eating the inner bark of *Pinus sylvestris*, both in the ethnographic literature and in our fieldwork, but no reports from Estonians living in the same area. Additonally, the shoots of pine trees were also mainly eaten by the Seto, while the shoots of another conifer, *Picea abies*, were eaten by Estonians. For the Seto, *P. abies* was considered to have too mild a taste. Although it is mentioned in contemporary literature that the cambium of *Populus tremula* was a widespread treat among Seto children in the spring [19], this was not confirmed in the archives, literature, or our fieldwork.

Differences related to childhood snacks were even more pronounced: When we asked separately about snacks, the Seto reported only eating *Trifolium* sp. flowers, while Estonians also ate the flowers of *Lamium album*, *Prunella vulgaris,* and *Primula veris*. Estonians also snacked on *Tilia cordata* buds, *Elymus repens* stems and roots, *Phragmites australis* spring root shoots, *Rosa* sp. and *Empetrum nigrum* fruits, and *Equisetum arvense* tubers. At the same time, the Seto have exclusively eaten *Pyrus pyraster* and *Malus sylvestris*, which may be due to the fact that those trees are only found in a few places in Setomaa and not in Võromaa.

Among the Seto, there were so-called collective plant names based on utilization. We were repeatedly told by interviewees that in their childhoods, their mothers referred to all herbs (both wild and cultivated) that were used to make tea by the common name “tsäihainad” (translated: “plants or grass that can make tea”). When asked to recall which particular species their mothers used, they indicated all those that were immediately gathered from the garden and the surrounding environment. Indeed, some species had “tsäihainad” as their only known name (e.g., *Trifolium repens*, *Trifolium montanum*, *Matricaria discoidea*). Our Seto interviewees repeatedly said that *Matricaria discoidea* tea used to be one of the most common recreational teas, but today only a few respondents drink it because the plant has disappeared from yards due to the current intense grass mowing. This tea has never been used among Estonians. Among the Seto, there was also a clear distinction between shop tea and herb tea, the latter of which was called “haina tee” (translated: “grass tea”): “Any plant which could be made into *tšai* (tea) my mum called ‘hainad’” (Seto man, born 1953). An important condition for the selection of “haina” (grass tea plants) was the pleasant smell: “There was no (black) tea in my childhood. / --- / Mother went to pick plants, and she never said their names to us. At that time, we didn’t even know that they were *angervaks*, *põdrakanep*, or those other plants. Mom just showed us the right flowers, and we picked them up. She collected all that smelled good, she said then the tea is good too” (Seto man, born 1958). Similar logic, in which the name of the plant is based on its usage, was also common in Pernau in pre-written South-Estonian (see [65]). An archival text written in the 1940s states that herbal teas began to be widely used in the Võromaa region in the late 19th and early 20th centuries [57]. This coincides with the time when the literature began to promote herbal teas [66].

### 3.2. Diachronic Evaluation (From End of the 19th Century to Today)

The historical use of wild food plants in the region covers only part of the uses that we recorded in this study (Table 2). Our analysis of the historical data shows that compared to the rest of Estonia, the most abundant reports on the use of wild plants during times of famine came from Võromaa and Setomaa, and a large number of the taxa used have not been reported elsewhere in the country. For example, in Rõuge and Põlva parishes, the seeds and aerial parts of *Calluna vulgaris* were crushed, mixed with flour, and made into a kind of cake (*karask*) during the famine of 1860–1870 [47]. The last remembered famine was in the 1860s, when several successive periods of unusual weather caused crop failure and starvation among peasants [67]. Although serfdom was abolished in Estonia in 1868, the archive has several records recalling that during the period of serfdom, food shortages and famine were particularly acute. Later, wild species were used temporarily during spring food shortages, such as in a commonly made hotpot with weeds called “naadiroog”, which resembles a thicker soup. When rye flour came to the end in the spring, a “naadikarask” (a cake of weeds) was made with non-fermented barley dough. The wild plants used for making both *naadiroog* and *karask* were approximately 15-cm-long spring shoots of various plants, most commonly *Cirsium arvense*, but also other wild taxa, which were not distinguished by name. The recipe for *naadikarask* was simple: One record from Räpina states that all kinds of plants were picked, such as *Cirsium* sp., young *Urtica dioica* and *Humulus lupulus*, *Ribes nigrum* and *Salix* sp. leaves, and spring chlorophyll-free shoots of *Equisetum arvense*, all finely cut and stewed until soft. This mass was then mixed with the pre-made *karask* dough and placed in the oven for baking. Wild species were also occasionally mixed with rye flour dough [47]. For *Humulus lupulus*, *Ribes nigrum*, and *Salix*, there are no reports of their use in the making of *karask* elsewhere in Estonia.

Moora [47] points out that people living near the border learned to use new plant taxa from Russians. For example, she cites a soup made with the leaves of *Arctium tomentosum*, which has not been mentioned elsewhere in Estonia, and a soup made with the leaves of *Tussilago farfara* and groats not reported elsewhere [47]. Yet, there might have been several other reasons for the high number of plants used during food shortages and famine in historical Võromaa and Setomaa. It is possible that the historical correspondents who returned information to the museum from this region may just have been more attentive and had written down all possible species and their usage. It is also possible that this area was one of the poorest areas of Estonia, where eating wild plant taxa during spring food shortages may have been more widespread than elsewhere. Therefore, those taxa might have had a so-called “famine food” image, and eating them was considered unusual in times of plenty, thus associating wild plants with hunger, whereas elsewhere in Estonia wild food plants were simply eaten for variety in taste. For example, *Rumex acetosa*, *Urtica dioica*, *Cirsium arvense*, *Aegopodium podagraria*, and some other plants were widely used as springtime foods all over Estonia, but in Setomaa and Võromaa they were associated with starvation.

It is remarkable, however, that as in the historical case of *naadikarask,* the same applies to the current use of wild food plants for soups or salads in the spring–the taxa are interchangeable. Many people told us they watch the growth of young grasses and picked all which reach a certain height and are distinguishable. Ethnic recipe books call such foods “keväjäne hainaruug” (spring grass hotpot) or “varakevadine heinasupp” (early spring grass soup) [22]. Such foods were made by both the Seto and Estonians. The soup is very simple: Washed and chopped “grass” is added to bone broth with cereals and potatoes at the end of cooking. “Grass” refers to the young leaves of *Urtica dioica*, *Aegopodium podagraria*, *Filipendula ulmaria*, *Primula veris*, and *Stellaria media* or other similar species. Often, a chopped boiled egg was added to the dish. The same plants, along with *Taraxacum officinale* leaves, are also currently harvested for “spring green salad”.

However, in our fieldwork, the negative image of eating wild plants as food during times of hunger was quite evident. It was quite common to receive an answer from an interviewee born in the 1960s along the lines of: “we were not so poor that we had to eat nettle, goutweed and gousefoot, we ate meat in our childhood”. This may be the reason why there are no longer any reported widespread wild plants that were used in the past like *Chenopodium album* and *Cirsium arvense*. It could also be because, as our interviewees said repeatedly, in large fields, intensive fertilization and poisoning took place during Soviet times as well as now. Additionally, instead of vegetable gardens, there are now ornamental and herb gardens, so there is literally no place for the weeds to grow.

Several other plant uses and customs that were no longer practiced elsewhere in Estonia were preserved in peripheral areas until relatively late. For example, Moora [51] says that while the fermented beverage made of juniper berries was formerly known all over the country, especially in western Estonia, by the 1930s it was only preserved in Setomaa and Russian villages near Lake Peipsi. Characteristically, in the area beer or *taar* (or near-bear) tare were made not from malt flour but from pre-baked malt cakes [51]. The use of birch sap in the area was historically very common, and the fermented sap was called by a variety of names: *taar, mahlataar, mahlajook, hapumahl, kali, mahlakali,* and *kasekali* [50]. Birch sap was fermented for at least 1–2 weeks with burnt rye flour or rye grain, and often with *vahaliim* (a light honey water obtained through the intense boiling of honeycombs in water); it was claimed to contain alcohol. It is known from Räpina parish that syrup was made by heating the sap, but it was not profitable to do so [50]. Today, local souvenir shops sell 100-mL glass bottles of birch sap syrup for ice cream and for marinating meat and 200 mL glass bottles of maple syrup for drinking made by small local producers (“Kasevetekohin”).

Moora [48] indicates one unique way of preserving berries specific to the region, which was not known elsewhere in Estonia. Namely, harvested and washed *Vaccinium vitis-idaea* berries were placed in a barrel along with *miiliim* (honey water) and then put under light press. Later, the berries were eaten along with the *miiliim* [48]. The *miiliim* was obtained by soaking the emptied honey combs in water. It is not clear from the article whether this was done during a sugar shortage or whether it was an archaic use. South-eastern Estonia has a historically very rich forest beekeeping tradition, and so it could be a method of preserving berries conceived locally.

### 3.3. Changed Borders and the Centripetal Effects

Being in the borderland played a great role in developing the habit of picking wild berries, which was historically more common here than elsewhere in Estonia. It may have been due to the influence of Russian culture, in which berry-picking was a long-standing tradition. As wild berries have good harvest years periodically, depending primarily on the weather, nature signs are read in early spring; for example, the Seto people believe that when heavy snow falls during butter week (8 weeks before Easter), a berry-rich summer is expected that year [68]. The greater harvest in the border area is due to the fact that elsewhere in the area wild berries were harvested mainly for personal use, whereas in the border area they were harvested to make a vital income. The picking of berries to sell was particularly prevalent when the Veriora railway station was opened along the Tartu-Pechory railway in the 1930s. As urban markets (in Tartu and Pechory) sold a kilogram of berries for more than four times the price paid to collectors or middlemen, the pickers themselves were also active in selling in the cities (especially *Vaccinium oxycoccos*, *Vaccinium vitis-idaea*, *Vaccinium myrtillus*, *Rubus chamaemorus*). Still, large quantities, amounting to hundreds of kilograms, were continuously sold to collectors who came to the villages [46,69].

During the Soviet era, there was a so-called women’s market at Pechory Monastery, where women sold vegetables, fruits, including berries, and canned homemade garden products [26].

“We went to the Pechory market here to sell berries, meat, potatoes, cucumbers, cabbages and other things. The price at the market was much higher than if we sold to a middleman in the Goods Office. For example, if a middleman in the Goods Office was buying for 10 kopecks a kilo, you could get 20 kopecks in the market. Although you could sell a large quantity, like a hundred kilos, to a middleman in the Goods Office, at the market you could sell only a few dozen kilos. The berries that sold the most at the market were: bilberry, cranberry, lingonberry–we do not grow more here.” (Seto man, born 1953).

The collecting of *Vaccinium oxycoccos*, the region’s most popular berry, was popularized by high buying-in prices during Soviet times. Planned state purchases under the Soviet economy began in the late 1970s and continued until the late 1980s. At that time, people in border areas took a boat across the lake to pick berries in Russian bogs, which was more convenient than going to Estonian bogs by car:

“We went there in Russia to pick berries. All the time, as the state border was still open. There were big swamps in Russia where we went to pick cranberries and cloudberries. From here, it was very easy for us to take a motorboat across the lake, only three kilometres away. During the Soviet era in the village, almost everyone had a boat. There are also large swamps in Meerapalu [Estonia] where many berries grow, but it’s a long way to get there by car, more than half an hour. Once upon a time, there were special dates on the Estonian side when one could start picking cranberries [every year]. On the Russian side, there were no restriction dates. This made it easier to go there to pick berries. Here on the Estonian side there were controls on the highway. The berries were confiscated from those who had previously visited the swamp and picked cranberries.” (Estonian woman, born 1939).

Berries were also brought from the other side of the administrative border for personal consumption:

“We have no cloudberries here. My wife is from Russia [Seto]. She knew where in Russia those berries grew. So, we went there a couple of times to pick those berries. Here in Estonia, there are also cloudberries in Meerapalu, but it is a very long way there. We haven’t been there to pick cloudberries.” (Seto man, born 1947).

On the Estonian side, the purchase prices of wild berries have been higher. In Soviet times, the Russians brought hundreds of kilograms of wild berries to Estonia:

“People from Russia’s villages brought huge amounts of cranberries to middlemen in the Goods Office in Värska [Estonia]. Both by car and by boat. People lived in the swamp for several days and harvested 15 or 20 kg a day. They had to pick berries one after the other, because otherwise someone would come and pick them themselves. They picked all the berries from one area. And then 200, 300, 600 kg were brought here at once.” (Seto man, born 1960).

This activity generally ceased in the 1990s. However, it continued to a limited extent in Saatse village, as a border crossing point for locals is located there, where one can cross the border on foot or by bike. Until recently, Russians and the Seto living on the Russian side of the border sold both wild berries and mushrooms in Estonia. Then, as we were told, after the raid on customs a couple of years ago (around 2017), the Estonia Customs Office banned the import of berries and mushrooms from this border point.

### 3.4. Resurgence and Commodification of Local Gastronomy

**Figure 5:**
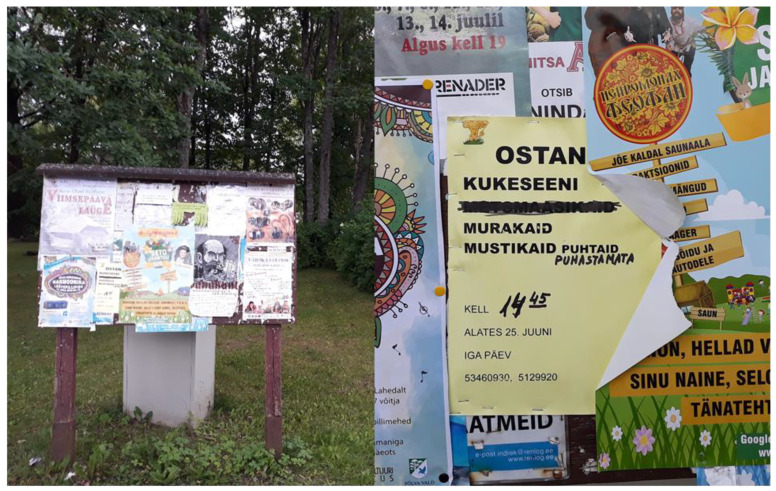
Message board in Orava village: “Every day from 25 June, I buy chanterelles, cloudberries, bilberries”. Photos: Renata Sõukand 7 July 2018.

The current commercialization of wild food plants is affected by market demand on the one hand and the distribution of subsidies to poor border areas (Setomaa Development Program, Border Area Development Program, etc.) on the other. Today, market demand is primarily concerned with the purchase of wild mushrooms and berries. During fieldwork, we observed numbers of buying announcements at the area’s bus stops, on the walls of small shops, and in the public advertising spaces of village centers (Figure 5). Middlemen with small vans drive around the villages two or three times a week during the season and stop at bus stops at particular times. There are several middlemen working in parallel. Wild mushrooms (mostly chanterelle) and wild berries are purchased. According to interviewees, it is mainly retired people who sell mushrooms to earn some extra income. Indeed, the region has a high proportion of older people. Working-age people, who live in rural areas, are already very busy. Thus, there is a severe labor shortage during the berry ripening and mushroom growing season. We were repeatedly told by interviewees that in Soviet times the forests were full of berry pickers and mushroom gatherers and there were not enough mushrooms or berries for everyone. Now the opposite is true, as forests and bogs are full of mushrooms and berries, but there are no pickers. However, the most recent high-turnover commercial purchase period lasted from the 1990s until the 2000s, when bilberries were bought in abundance. As for today (2018–2019), it was repeatedly said by interviewees that now it is not worth going to pick berries for personal consumption because the long journey does not pay off, and in the past, it only paid off if most of the berries were sold. It was also said that the use of bilberries in particular has been influenced by intensive forest management since the early 2000s. Because of this, old forests suitable for bilberries have been cut down and there are simply no more berries in the areas where people used to pick them. Similar results have been obtained from a recent nationwide survey of bilberry pickers. It turned out that berry-pickers preferred the public forests familiar to them to protected areas, whereas 60% of respondents said that they experienced the loss of berry sites due to clear-cutting [70].

Within the last five years, in their search for a niche, small producers in the region have begun to appreciate raw materials from nature. For example, the Nopri Farm Dairy, which we visited during participant observation in the field, started bottling pasteurized and also fermented birch sap a few years ago. However, this is a so-called market test and it is not yet known how the market will adopt this rather expensive product in the long run. Of the wild plants, clover leaves were still used, as they were added to cheese for taste. However, from where the herb is obtained, the owner did not tell us. The biggest problem now, said the owner of a cow farm, is the lack of labor in the region. Today, he hires workers from Ukraine.

**Figure 6:**
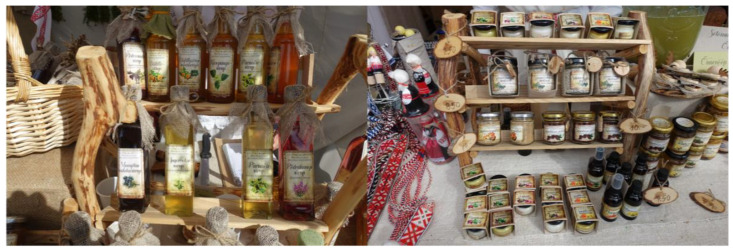
Seto Kingdom Day on 5 August 2018 in Lüübnitsa village. A selection of products from small local producers in Setomaa. Kingdom Day is one of the most important events in the region where home-produced products are traded. Photos: Renata Sõukand.

We also saw wild berry wines in addition to horticultural wines (strawberry, rowan, apple, red currant, marigold, etc.) at one of the most progressive home restaurants in the region, Maagõkõnõ, in Saabolda village. However, their *Viburnum opulus* wine, for example, has never been made in the region, either historically or today, based on our fieldwork. However, both Nopri Farm and Maagõkõnõ sell the region’s special product, namely raw cheese (*sõir*) that is seasoned with *Carum carvi* seeds. However, cumin is no longer picked locally but rather bought in the shop. There are active workshops in the area to prepare different food products. For example, Räpina Creative House offers home wine courses and makes wild berry wines, such as cranberry, which are sold at local fairs. One of the biggest tourism and identity events in the area is the annual Seto Kingdom Day in August. This is the only place where so-called semi-officially sold home-made vodka, *handsa*, can be found. In 2019, for example, one producer sold “handsa” flavored with *Vaccinium myrtillus* berries. This drink is also mentioned in old archival texts. Additionally, in 2018 and 2019, various handmade products were on sale (Figure 6): Pine cone jam; juniper twig syrup, and sea-buckthorn berries in juniper syrup; juniper berry salt and mustard; dandelion flower honey, dandelion syrup and dandelion syrup with rhubarb; rosebay willowherb flower syrup and meadowsweet flower syrup; cowberry jam with apple and pear; spruce needle honey; and birch bud syrup and herbal mixtures for tea. Tourism has therefore created new site-specific products and also helps to keep traditional products alive.

## 4. Conclusions

On the basis of the historical data, we can say that the border area became herbophilic earlier than the rest of Estonia. This might be due to cultural contact with neighboring Russians, who may have taught, for example, the use *Arctium tomentosum* and *Tussilago farfara*, but also to the fact that the region is the poorest in the country. Yet, this has not continued to the present. When the border closed in the early 1990s, the city of Pechory, which was historically the center of attraction in the region, lost its importance. This brought about a decline in the exchange of knowledge as well as commercial activities around wild food plants, as horticultural products and wild berries were sold there. National support for businesses in the area today and also mass cultural events have, on the other hand, introduced new wild food plant applications and are helping to preserve local plant-specific uses in the area.

The Seto have still preserved their distinctive features either by consciously opposing others or by maintaining more historical plant uses. The inhabitants of Setomaa and Võromaa associated wild plants with famine food in the early 20th century, yet it was stressed more now by the Seto than by Estonians. Along with the popularity of a healthy lifestyle, the use of new plants is now based more on examples provided by people within the community (e.g., more influenced by local authorities like schoolteachers) than those directly listed in the literature. The Seto, who have lived for centuries in a more closed cultural space, have tried fewer wild plants as a snack. Estonians who are more open to other cultures have also been more eager to experiment with wild plants.

Prior reforms, such as the collective farms established during the Soviet era, have lost their importance due to several recent changes. The transition from a planned economy to a market economy when the former network of points of purchase ceased to exist also had a major impact. However, the closure of the border in the 1990s has had less impact on the use of wild species in border areas than the change and loss of the natural environment. Thus, *Vaccinium myrtillus* has begun to disappear in the past few decades due to intensive clear cutting. In contrast, some species have been affected by intensive mowing of yards and gardens (e.g., *Matricaria discoidea*) and by the overgrowth of open areas (e.g., *Thymus serpyllum*). The combination of several factors has led to the disappearance of *Carum carvi*. Influenced by literature, people started to experiment with previously unused plants (e.g., *Filipendula ulmaria*, *Epilobium angustifolium*). While earlier there was a need to earn a living by harvesting wild berries, nowadays, the standard of living has risen to such an extent that driving tens of kilometers away to marshes to collect *Vaccinium oxycoccos* is no longer worthwhile.

Future research in the region should record the numerous innovative wild products sold at fairs but not used locally. Only the expansion of narrowly targeted tourism products can secure their success in marketing, yet as it is strongly related to seasonality, the spring-summer tourist season is not enough to sustain the practice of using wild food plants, which is an additional safeguard for the food security of the local population in potential times of crisis. This requires the maintenance of customary practices, i.e., to value “simple work”. The common current practice of picking forest berries to sell is kept alive mainly by older individuals, while young people already have the habit of buying berries at the shop rather than picking them themselves, and this is simply not sustainable.

## Figures and Tables

**Table 1:** Demographic information about the interviewees.

Language Group	Seto		Võro, Estonian	
**Gender**	F	M	F	M
Number of people	22	15	19	16
Total number of people	37		35	
Mean age	70	66	68	69
Religions	Orthodox mixed with paganism/non-believer		Lutheran/Orthodox/non-believer or own religion (mixed religion or tradition/nature believer)	
Educational composition	Proportionally divided between primary, secondary and high school and university-level education.			
Origin	Local Setos and Setos who earlier lived on the Russia side of the border		Local non-Setos living in the territory of historical Setomaa and Võromaa.	
Languages spoken by parents	Seto		Mostly Võro, less than 10% Tartu dialect or one of the parents a Russian speaker.	

**Table 2:** Wild food plants use by Setos (*n* = 37)/Estonians (*n* = 35).

Latin Name	Local Name (Plural)	Parts Used	Preparation	Food	Past Uses (DUR)			Current Uses (DUR)	
					CH	AB	AD	NW	AT
Adoxaceae									
*Viburnum opulus* L.	Lodjapuu ^B^, lodjappuu ^B^	Fruits	Frozen	Eaten ^V^					
Amaranthaceae									
*Chenopodium album* L.	Malts ^A^, hanemalts ^A^	Young plants	Cooked	Soup ^V^					
Apiaceae									
*Aegopodium podagraria* L. (UVV.EB:SE108)	Naat(id) ^A^, varesjalg ^B^	Young leaves	Cooked	Omelette			/1		
				Soup ^V^			1		
				Famine food ^S^					
			Fresh	*Suuliim*				1/1	
				Snack				/1	
				Salad			5	1/2	
*Angelica sylvestris* L.	Tjägil ^NF^, pütsk ^B^, pujokõnõ ^NA^	Roots	Dried	Additive to vodka	/1				
		Stems	Fresh	Snack ^V^	1				
*Carum carvi* L. (UVV.EB:SE31)	Köömen (ed) ^A^, küümen (ed) ^B^, küümne (d) ^B^	Seeds	Dried	Additive to red beetroot salad	1				
				Additive to sauerkraut	/1	3/11			1
				Seasoning for fish					/1
				Seasoning for curds ^S^	1/1				
				Seasoning for food ^V^	1				
				Seasoning for bread ^S, V^	3/3				
				*Sõir*	7/6	2/2	/1	1	1
				Tea ^S, V^	4/8	1/4	/1		2/4
				Seasoning for white bread ^V^					
Asparagaceae									
*Maianthemum bifolium* (L.) F.W.Schmidt	Varsakabja ^B^	Fruits	Fresh	Snack ^V^					
Asteraceae									
*Achillea millefolium* L. (UVV.EB: SE062, SE077, SEDR009)	Raudrohi ^A^	Inflorescences	Dried	Tea				2/1	
*Artemisia absinthium* L.	Pälünä ^B^, pälüna ^B^, pännüla ^B^	Inflorescences	Dried	Tea ^S^					
				Seasoning for *taar* ^V^					
*Arctium tomentosum* Mill.	takjas ^A^	Leaves	Cooked	Soup ^Vk, Sk^					
*Cirsium arvense* (L.) Scop.	Põldohakas ^A^, naadid ^NA^, ohtkja ^B^, ohtja ^B^, ohaka ^A^, põldohtjas ^NA^	Young plant	Cooked	Soup ^V^					
				Famine bread ^S, V, Vk^					
*Matricaria discoidea* DC. (UVV.EB: SEDR002, SEDR019, SE017, SE066, SE043, SE005)	Murukummel ^NF^, morokummel ^NF^, tsäihain ^NF^, ubinhain ^B^, upinhain ^B^, kummel ^A^, hoovikummel ^NA^, ubinhein ^B^, upinhein ^B^	Aerial parts	Dried	Tea ^S, V^	14/4	4	1/2		3/1
		Flowers	Dried	Tea					1
*Taraxacum officinale* F.H.Wigg. s.l. (UVV.EB: SE107)	Võilill ^A^	Flowers	Cooked	“Honey”		2/3	2/2		
			Fresh	Snack			1		/1
		Leaves	Fresh	Salad			3	/2	
		Leaves	Fresh	Snack			1		
		Roots	Fresh	Snack			1		
		Roots	Roasted	Coffee substitute		/1	1		
*Tussilago farfara* L.	paiseleht	Leaves	Cooked	Soup ^Vk^					
Betulaceae									
*Alnus glutinosa* (L.) Gaertn.	Must lepp ^A^	Wood	Burned	Meat and fish smoking					/1
*Alnus incana* (L.) Moench (UVV.EB: SE121)	Hall lepp ^A^, tavaline lepp ^NF^, valge lepp ^A^, lepp ^A^	Cambium	Fresh	Snack ^S^	/4				
		Wood	Burned	Meat and fish smoking	1	2/3			/6
				meat smoking	/1	3/6			3/1
			Burned to charcoal	Clean homemade vodka		2			
		Wood and leaves	Burned	Meat and fish smoking					1/1
				Meat smoking		1			1
*Betula* spp. (incl. *Betula pendula* Roth & *B. pubescens* Ehrh.) (UVV.EB: SE117, SE076)	Kask ^A^, kõo ^B^, kõivo ^NF^, kõiv (u) ^B^	Buds	Dried	Tea			1	/1	
		Cambium	Fresh	Snack ^S^	2/4				
		Leaves	Dried	Tea	1				
		Sap	Fermented	Drink ^V^	6/5	5/12			9/4
				*Suuliim*	1				
				*Taar*	/1				
			Fresh	Drink ^S, V^	4/4	4/7			14/15
			Frozen	Drink				/6	2
			Boiled	Syrup ^V^					
		Wood	Burned to charcoal	Clean homemade vodka		1			
*Betula pendula* Roth	Aro kõiv ^B^, mahla kõiv ^NA^	Sap	Fresh	Drink ^V^					
*Corylus avellana* L. (UVV.EB: SEDR015, SE018)	Pähkel ^A^, sarapuu ^A^	Seeds	Dried	Snack ^S^	2/4	1/4	2/1		2/2
				Milled for cakes ^V^					
Bryophyta									
Bryophyta	Sammal ^A^	Aerial parts	Dried	Famine bread ^S^					
Cannabaceae									
*Humulus lupulus* L. (UVV.EB: SE028)	Humal ^A^	Cones	Fermented	Additive to beer ^V^	2/3	/1	1/1		
				Additive to taar ^V^					
		Young plant	Cooked	Famine bread ^Vk^					
Caprifoliaceae									
*Valeriana officinalis* L. (UVV.EB: SE123)	Palderjan ^A^	Roots	Dried	Additive to vodka ^V^		1			
		Flowers	Fresh	Additive to vodka ^V^					
Caryophyllaceae									
*Stellaria media* (L.) Vill. (UVV.EB: SE069)	Nätsern ^B^, vesihein ^A^	Aerial parts	Fresh	Salad			1		
Cupressaceae									
*Juniperus communis* L. (UVV.EB: SE116)	Kadakas ^A^, kadaja ^B^, kattai ^B^	Fruits	Dried	Tea	1/1				
			Fermented	*Kali* ^S, Vk, Sk^	1				
				*Taar* ^S, V, Vk, Sk^	6	2			
				Beer ^V, Vk, Sk^					
			Fresh	Additive to fermented birch sap	1				1
				Marinade for game meat				1	
				Snack	1		/1	1	3/4
		Twigs	Burned	Meat smoking	1	/1			1
			Fermented	Filter for *taar*	1				
				*Taar*	1				
			Fresh	Additive to *kali*	/3	1			
			Fermented	Filter for beer		1			
Cyperaceae									
*Schoenoplectus lacustris* (L.) Palla (UVV.EB: SE053)	Pütsk ^NF^, luga ^B^, jõeluga ^NF^, plukna ^NF^, lug’n ^NF^	Lower end of white stem	Fresh	Snack	4/4				
Dennstaedtiaceae									
*Pteridium aquilinum* (L.) Kuhn	Käpiline sõnajalg ^NA^, sõnajalg ^A^	Roots	Cooked	Famine bread ^S^					
		Leaves	Cooked	Famine bread ^S, V^					
	Equisetaceae								
*Equisetum arvense* L. (UVV.EB: SE020, SE088)	Põldosi ^A^, maapähkel ^NF^	Tubers	Fresh	Snack	/1				
		Young plant	Cooked	Famine bread ^Vk^					
Ericaceae									
*Arctostaphylos uva-ursi* (L.) Spreng.	Leesikas ^A^	Leaves	Dried	Tea	1				/3
*Calluna vulgaris* (L.) Hull	Kanarbik ^A^	Aerial parts	Dried	Famine bread ^Vk^					
*Empetrum nigrum* L. (UVV.EB: SE110)	Karumustikas ^NF^	Fruits	Cooked with *Vaccinium myrtillus*	Jam					/1
			Fresh	Snack					/1
*Ledum palustre* L.	Sookikka ^B^, sookail ^A^	Flowers	Fresh	Additive to *taar* ^V^					
*Vaccinium myrtillus* L. (UVV.EB: SE021)	Mustikas (d) ^A^, mustik ^B^, mustk’ ^B^, mustka ^B^	Aerial parts	Dried	Tea ^V^	1/2		/1		2
		Flowers	Dried	Tea ^S^					
		Fruits	Cooked	Compote		1/1			/2
				Jam ^V^	3/4	14/11	1/1		7/9
				Jam without sugar	2				
				Kissel	1/2	1	1		3/2
				Lemonade					1
			Dried	Snack		3			
				Additive to vodka ^S, V^					
			Fermented	Wine ^V^			1		
			Fresh	Pie	1/2	1	1	1	1
				Dessert	1	1		1	/1
				Snack ^V^	2/4	7/7	/1		26/22
			Frozen	Raw jam		/1	1	6/7	
				Food		2	1/1	3/6	
			Jam	Lemonade		/1			
				Tea	1				
			Dried	Tea					1
			Stored in water	Snack	1	/1			
			Steamed	Juice		/1			
*Vaccinium oxycoccos* L. (UVV.EB: SE109)	Jõhvikas (d) ^A^, kuremari (ad) ^B^, kuremara’ ^B^, kurõmari (’a) ^NF^, kurõmarä ^NA^	Fruits	Cooked	Compote		/1			
				Jam	1/1	7/3			3/2
				Juice	1	5/1	/1		2/4
				Kissel	1/3	7/5	/1		5/1
				Semolina foam		2/2	/2		2
			Fermented	Additive to sauerkraut ^V^	3/3	3/6			
				Wine			1/1		
			Fresh	Additive to fermented birch sap	1	1			
				Lemonade	1/1	4/3			3/1
				Snack ^V^	1/2	12/12	/1		21/12
				Sugar candies		1	1		1
				Tea					/3
			Frozen	Raw jam				/1	
				Food			1/1	2/3	1
			Macerated in vodka	Homemade liqueur			/4		
			Stored in water	Snack	1/3	5/6	/1	1	4
*Vaccinium uliginosum* L. (UVV.EB: SE036)	Joovikad ^B^, joovhke ^NA^, sinikas (d) ^A^, karumustikad ^NF^	Fruits	Cooked	Jam	/1				/1
			Fresh	Snack ^V^	4/4				2/2
			Fermented	Wine ^V^					
*Vaccinium vitis-idaea* L. (UVV.EB: SE035, SE127)		Aerial parts	Dried	Tea ^V^	4/1		1		
	Pohl (ad) ^A^, palukas (d) ^B^, palohkas (d) ^B^	Flowers	Dried	Tea ^S, V^		/1			
		Fruits	Cooked	Compote		/1			
				Jam ^V^	1/5	11/9	/2	1	3/11
				Jam without sugar		1		1	
				Juice				1	
				Kissel		1	/1		2/1
				Lemonade					1
			Fresh	Toppins to ice cream				1	
				Pie				1	
				Snack ^V^	/3	8/12	1/1		18/20
				Raw jam	1		/1		
			Frozen	Raw jam				1	
				Food			/1	4/6	
			Jam	Additive to red beetroot salad	/1				
				Lemonade		/1			/1
				Tea	1		/1		
			Stored in water	Snack	1/1	2			
		Leaves	Dried	Tea					1/3
Fagaceae									
*Quercus robur* L. (UVV.EB: SE100)	Tamm ^A^	Acorns	Roasted	Coffee substitute ^V^			/1		
		Acorns	Dried	Additive to *taar* ^V^					
		Bark	Fresh	Additive to homemade vodka		3	/3		
		Leaves	Fresh	Additive to fermented cucumbers ^V^		1			2
Grossulariaceae									
*Ribes nigrum* L.	Must sõstar ^A^, sitke ^B^	Fruits	Fresh	Snack ^V^					/1
		Buds	Fresh	Smoothie				/1	
		Leaves	Dried	Tea				/1	
			Cooked	Famine bread ^Vk^					
Hypericaceae									
*Hypericum* spp. (incl. *Hypericum perforatum* L. & *H. maculatum*) (UVV.EB: SE059, SE002, SE003, SEDR012, SEDR028, SEDR020, SEDR031)		Aerial parts	Dried	Tea	2	3	1/1	1/1	/1
	Naistepuna ^A^	Flowers	Fresh	Salad			/1		
Lamiaceae									
*Lamium album* L.	valgete õitega nõges ^NF^, mesinõges ^NF^, piimanõges ^A^	Flowers	Fresh	Snack, flower sucked	/3				
*Mentha aquatica* L.	Piparmünt ^NF^, metsik piparmünt ^NF^	Aerial parts	Dried	Tea			1	/1	
*Mentha arvensis* L.	Piparmünt ^B^, põldmünt ^A^	Aerial parts	Dried	Tea			/1		
*Origanum vulgare* L. (UVV.EB: SEDR032, SE082, SE090)	Pune ^A^, mets-majoraan ^NF^, majoraan ^NF^, vorstirohi ^A^, maioroon ^NA^	Aerial parts	Dried	Seasoning for blood pudding ^V^	/1	/5			
				Seasoning for food ^V^					
*Prunella vulgaris* L. (UVV.EB: SE067, SE081)	Käbihein ^A^	Flowers	Fresh	Snack, flower sucked	/1				
*Thymus serpyllum* L. (UVV.EB: SE037, SEDR008)	Nõmm-liivatee ^A^, jaanihain ^NF^, jaanilill ^NF^, liivatee ^A^, lilla jaanihain ^NF^, maarjaheina ^B^, kolmekõrraline rohi ^B^, tiimjas ^NA^	Aerial parts	Dried	Tea ^S,V^	4		1		4
				Seasoning for sausage ^V^					
Leguminosae									
*Lotus corniculatus* L.	Virapool ^B^	Aerial parts	Dried	Tea ^S^					
*Trifolium hybridum* L.	Roosa ristikhein ^NF^	Flowers	Fresh	Snack, flower sucked		1			
*Trifolium montanum* L.	Tsäihain ^NF^	Aerial parts	Dried	Tea	1				
*Trifolium pratense* L. (UVV.EB: SE085)	Ristik ^A^, ristikhein ^B^, ristikhaani ^B^, punane ristik ^A^	Flowers	Fresh	Snack, flower sucked	3/5				
			Dried	Tea ^S,V^					
*Trifolium repens* L. (UVV.EB: SE032)	Maa-ristikhein ^NF^, maa-ristik ^NF^, valge ristikhein ^NF^, valge ristik ^A^, tsäi hain ^B^	Flowers	Dried	Tea ^S^			1/1		
			Fresh	Snack, flower sucked	/3	/1			1
Malvaceae									
*Tilia cordata* Mill. (UVV.EB: SE087, SE006, SEDR007, SEDR023, SEDR025, SEDR030, SEDR040)		Buds	Fresh	Snack				/1	
	Pärn ^A^, lõhmus ^A^, pähn ^B^, pähnapuu ^B^	Flowers	Dried	Tea ^V^	2	1	2/1	1	14/17
		Catkins	Fresh	Tea ^V^					
		Young twigs	Fresh	Tea ^V^					
**Oleaceae**									
*Fraxinus excelsior* L.	Saar ^A^	Wood	Burned	Meat smoking		/1			
Onagraceae									
*Epilobium angustifolium* L. (UVV.EB: SE049, SEDR034, SEDR036, SEDR037)	Põdrakanep ^A^, ivan-tšai ^NF^, pajulill ^A^	Flowering tops	Dried	Tea	1		/1	4	
		Leaves	Fermented	Tea				/1	
				Salad				/1	
Oxalidaceae									
*Oxalis acetosella* L. (UVV.EB: SE118)	Jänesekapsas ^A^, jäneshein ^NF^, oblikas ^NF^, hapuoblikas ^NF^	Leaves	Fresh	Snack ^V^	7/8	/2			1/4
				Soup ^V^	/1				
Pinaceae									
*Picea abies* (L.) H.Karst. (UVV.EB: SE126)	Kuusk ^A^, kuus ^B^	Resin	Fresh	Snack	/2				
		Shoots	Dried	Pie	/1				
				Tea				/1	
			Fresh	Snack	1/1		1	1/1	/2
				Taste-butter				1	
			Frozen	Smoothie				/1	
*Pinus sylvestris* L. (UVV.EB: SE120)	Mänd ^A^, pedaja ^B^, pedäja ^B^, pettäi ^B^	Cambium	Fresh	Snack ^S^	16				
		Shoots	Cooked	Syrup				1/1	
			Dried	Tea ^V^	1				
			Fresh	Snack	20/2				1
Poaceae									
*Elymus repens* (L.) Gould		Roots	Cooked	Food			/1		
	Orashein ^A^		Fresh	Snack	/1				
		Stems	Fresh	Snack	/2				
*Phleum* spp. (incl. Phleum pratense L. & P. pratense subsp. bertolonii (UVV.EB: SE124)	Timut ^A^, timmut ^B^, timmat ^B^	Stems	Fresh	Snack					1/2
*Phragmites australis* (Cav.) Trin. ex Steud. (UVV.EB: SE091)	Pilliroog ^A^	Spring root shoots	Fresh	Snack	/1				
Poaceae	Kõrrelised ^A^, kastehein ^A^	Stems	Fresh	Snack	3/5				1
Polygonaceae									
*Rumex acetosa* L. (UVV.EB: SE102, SE024, SE009)	Hapuoblikas (d) ^A^, oblikas (d) ^A^, hapuhain ^B^, hapuhein ^B^, hublikhain ^NF^, hublikas (d) ^NF^, hublik ^B^	Leaves	Cooked	Kissel	1				
				Soup ^V^	5/4	3/1	2/1	/1	4/2
				Famine food ^S^					
			Fresh	Salad				/1	
				Snack	13/13	5/2	/1		6/7
Primulaceae									
*Primula veris* L. (UVV.EB: SEDR003, SEDR039, SEDR042)	Nurmenukk ^A^, kikkapüks ^B^	Flowers	Dried	Tea			3/1	/1	2/2
			Fresh	Snack, flower sucked					/1
		Leaves	Fresh	Salad				/3	
Rosaceae									
*Alchemilla vulgaris* auct. (coll.) (UVV.EB: SE080)	Kortsleht ^A^	Leaves	Dried	Tea			/1		
*Amelanchier spicata* (Lam.) K.Koch (UVV.EB: SE019)	Poola kirss ^NF^, mets-aroonia ^NF^, saksa-toomingas ^NF^	Fruits	Cooked	Jam			1/1		
			Fresh	Snack	/1		3/1		/1
*Filipendula ulmaria* (L.) Maxim. (UVV.EB: SE041)	Angervaks ^A^	Flowers	Dried	Tea	1			/2	
*Fragaria vesca* L. (UVV.EB: SE022, SE036, SE086)	Maasikas (d) ^A^, metsmaasikas (d) ^A^, maasik (a) ^A^, maask ^B^, maaska ^B^, mõtsamaasik ^B^, mõtsmaasik ^B^, mõtsamaasiga ^B^	Aerial parts	Dried	Tea ^V^		1/1	1	/1	
		Fruits	Cooked	Jam	/4	1	/1		/4
			Dried	Tea	/1				
			Fresh	Dessert	3/2	7	/1	1	/1
				Pie	/1				1
				Snack ^V^	5/9	7/3	1/1	1	13/15
			Frozen	Raw jam		2	/2	3/7	/1
			Jam	Pie	/1				
		Flowers	Dried	Tea ^S, V^					
*Malus domestica* Borkh.	Õunapuu ^A^, kitseuibu ^NF^	Fruits	Fresh	Dessert	/1				
*Malus sylvestris* (L.) Mill.	Mõtsk ^NF^, metsik õunapuu ^NF^	Fruits	Frozen	Snack	1				
*Prunus padus* L. (UVV.EB: SE013)	Toomõ, toom ^A^, toomingas ^A^, toomepuu ^A^, toomik ^NF^, toomõkõnõ ^NF^, tuum ^B^	Fruits	Fresh	Snack ^V^	5/6	2	/1		2
			Raw jam	Pie	1				
		Leaves	Fermented	Additive to fermented cucumbers ^S^					
*Pyrus pyraster* (L.) Burgsd.	Kruusa ^NF^, prusa ^NF^	Fruits	Frozen	Snack	2				
Rosa sp. (Rosa majalis Herrm. & R. rugosa Thunb.) (UVV.EB: SE139)	Kibuvits ^A^	Fruits	Dried	Tea			1/3		/2
			Fresh	Snack				/1	/2
*Rubus caesius* L.	Põldmari ^A^	Fruits	Fresh	Snack			/1		
*Rubus chamaemorus* L. (UVV.EB: SE111)	Murakas (d) ^A^, murel ^NF^, murahka ^B^	Calyx	Dried	Tea					/1
		Fruits	Cooked	Compote	/1	/1			
				Jam	/1	/5	/2		1/2
			Fresh	Snack ^S^		2/2	1/3		3/5
			Frozen	Raw jam				/1	
			Macerated in vodka	Homemade liqueur			/1		
*Rubus idaeus* L. (UVV.EB: SE027, SEDR029b)	Vaarikas (d) ^A^, metsvaarikas (d) ^A^, vabarna (d) ^A^, vavarna ^B^	Fruits	Cooked	Compote		/1			
				Jam	1/3	6/4	3/2		1/7
				Juice		/1			
				Kissel					1
			Raw jam	Pie	1				
			Fresh	Snack ^V^	3/4	5/4	1/1	/1	6/8
			Frozen	Raw jam			/1	1/8	
			Jam	Pie	/1				
			Fresh	Dessert			/1		
			Dried	Tea ^V^					
		Leaves	Fresh	Additive to fermented cucumbers		2			
		Stems	Dried	Tea ^V^	3/5	3	1/1		
				Coffee substitute ^S, V^					
			Fresh	Tea	/2	/1			/1
		Stems with leaves	Dried	Tea	1/1				
		Flowers	Dried	Tea ^V^					
*Rubus nessensis* Hall (UVV.EB: SE040)	Tsiavabarna ^B^, tseavabarna ^B^, metsvabarna ^NF^, põldmari ^A^, põldmurakas ^NF^, kahruvabarna ^B^, karuvaarikas ^B^, must vaarikas ^A^	Fruits	Cooked	Jam		1	3		2
			Fresh	Snack	1	2	/1		8/2
			Frozen	Raw jam				1	
*Rubus saxatilis* L.	Linnohka ^B^	Fruits	Fermented	Wine ^V^					
*Sorbus aucuparia* L. (UVV.EB: SE026)	Pihlakas ^A^, pihlapuu ^A^, pihl ^A^, pihelgas ^A^	Fruits	Cooked	Compote		/1	1	/1	
				Jam	3	1	1/2		
			Fresh	Juice		/3			
			Dried	Snack				1	
				Tea	2/1		/1	1	1
			Fermented	Wine ^V^	1/2	1/3	/2		
				*taar* ^V^					
			Fresh	Additive to marinated cucumbers		/1			
				Pie	1				
				Snack ^S^	12/4	6/9	2/3		6/4
				Additive to vodka ^S,V^					
			Frozen	Snack ^V^	1/2	2			/2
		Flowers	Dried	Tea ^S, V^					
**Salicaceae**									
*Populus tremula* L. (UVV.EB: SE104)	Haab ^A^	Wood	Burned	Meat smoking			1		
*Salix* spp. (incl. *Salix cinerea* L., *S. aurita* L., *S. myrsinifolia* Salisb., *S. triandra* L. and their hybrids) (UVV.EB: SE044, SE030, SE083)	Paju ^A^	Cambium	Fresh	Snack	3/4				
		Leaves	Cooked	Famine bread ^Vk^					
Sapindaceae									
*Acer platanoides* L. (UVV.EB: SE125)	Vaher ^A^	Sap	Fermented	Drink			/1		
			Fresh	Drink ^V^	1/3	4/8	2/2		1/7
			Frozen	Drink			1/3		
		Catkin	Fresh	Tea ^V^					
Urticaceae									
*Urtica dioica* L. (UVV.EB: SE074)	Nõges ^A^, kõrvenõges ^A^, raudnõges ^A^	Young aerial parts, leaves	Cooked	Soup		/1			/1
				Famine bread ^Vk^					
			Burned	Fish smoking			1/1		
				Meat smoking		1/1			/3
			Dried	Cutlet			1		
				Seasoning for food			/1		
				Tea			3	/1	
			Fresh	Omelette				1/1	
				Pan-fried				/1	
				Salad			2	1/1	
				Soup ^V^	3/5	2/2	4/4	1/2	5/1
				*Suuliim*				1/1	
				To keep crayfish alive		/1			

Abbreviations: AB–abandoned in adulthood, AD–temporary used in adulthood, AT–always used, CH–abandoned in childhood, NW–learned only recently (within the last 5 years). Historical data: archive (1880s to 1960s) ^(S,V)^ and ethnographic literature ^(Sk,Vk)^ (^S^ = historical Setomaa; ^V^ = historical Võromaa (Räpina, Vastseliina, Rõuge, Põlva–parishes)) that has studied this period and region [46,47,48,49,50,51]. Plant name analysis based on [58]: ^A^ = plant name spread all across Estonia, ^B^ = dialect plant name (Seto and Võro), ^NF/NA^ = new plant name from field work/archive. *Suuliim* is a traditional cold soup or cold sauce. *Kali* and *taar* are traditional non-alcoholic fermented beverages. *Sõir* is a traditional non-fermented cottage cheese.

## References

[1] Leonti M., Nebel S., Rivera D., Heinrich M. (2006). Wild gathered food plants in the European Mediterranean: A comparative analysis. Econ. Bot..

[2] Rivera D., Obon C., Heinrich M., Inocencio C., Verde A., Fajardo J., Heinrich M., Müller W.E., Galli C. (2006). Gathered Mediterranean food plants–ethnobotanical investigations and historical development. Local Mediterranean Food Plants and Nutraceuticals.

[3] Jönsson H., Lysaght P., Jönsson H., Burstedt A. (2013). The Road to the New Nordic Kitchen—Examples from Sweden. The Return of Traditional Food. Lund Studies in Arts and Cultural Sciences.

[4] Larsen H., Österlund-Pötzsch S., Lysaght P., Jönsson H., Burstedt A. (2013). Foraging for Nordic wild food introducing Nordic island terroir. The Return of Traditional Food. Lund Studies in Arts and Cultural Sciences.

[5] Neuman N., Leer J. (2018). Nordic Cuisine but National Identities. “New Nordic Cuisine” and the gastronationalist projects of Denmark and Sweden. Anthropol. Food.

[6] Kalle R. (2017). Change in Estonian Natural Resource Use: The Case of Wild Food Plants. Ph.D. Thesis.

[7] Sunderland T.C. (2011). Food security: Why is biodiversity important?. Int. For. Rev..

[8] Schulp C.J., Alkemade R., Klein Goldewijk K., Petz K. (2012). Mapping ecosystem functions and services in Eastern Europe using global-scale data sets. Int. J. Biodivers. Sci. Ecosyst. Serv. Manag..

[9] Peciña M.V., Ward R.D., Bunce R.G., Sepp K., Kuusemets V., Luuk O. (2019). Country-scale mapping of ecosystem services provided by semi-natural grasslands. Sci. Total Environ..

[10] Lõhmus A., Remm L. (2017). Disentangling the effects of seminatural forestry on an ecosystem good: Bilberry (Vaccinium myrtillus) in Estonia. For. Ecol. Manag..

[11] Hadjichambis A.C., Paraskeva-Hadjichambi D., Della A., Elena Giusti M., De Pasquale C., Lenzarini C., Censorii E., Reyes Gonzales-Tejero M., Patricia Sanchez-Rojas C., Ramiro-Gutierrez J.M. (2008). Wild and semi-domesticated food plant consumption in seven circum-Mediterranean areas. Int. J. Food Sci. Nutr..

[12] Pardo-de-Santayana M., Tardío J., Blanco E., Carvalho A.M., Lastra J.J., San Miguel E., Morales R. (2007). Traditional knowledge of wild edible plants used in the northwest of the Iberian Peninsula (Spain and Portugal): A comparative study. J. Ethnobiol. Ethnomed..

[13] Maffi L., Woodley E. (2012). Biocultural Diversity Conservation: A Global Sourcebook.

[14] Ghirardini M.P., Carli M., Del Vecchio N., Rovati A., Cova O., Valigi F., Agnetti G., Macconi M., Adamo D., Traina M. (2007). The importance of a taste. A comparative study on wild food plant consumption in twenty-one local communities in Italy. J. Ethnobiol. Ethnomed..

[15] Nebel S., Heinrich M. (2009). Ta chòrta: A comparative ethnobotanical-linguistic study of wild food plants in a graecanic area in Calabria, Southern Italy. Econ. Bot..

[16] Kalle R., Sõukand R. (2016). Current and remembered past uses of wild food plants in Saaremaa, Estonia: Changes in the context of unlearning debt. Econ. Bot..

[17] Sõukand R., Kalle R. (2016). Perceiving the biodiversity of food at chest-height: Use of the fleshy fruits of wild trees and shrubs in Saaremaa, Estonia. Hum. Ecol..

[18] Sõukand R. (2016). Perceived reasons for changes in the use of wild food plants in Saaremaa, Estonia. Appetite.

[19] Hagu P., Hõrn A., Lillak A., Kalkun A., Külvik H., Nassar E., Pajusalu K., Tiideberg K., Vissel K. (2014). Setomaa: Unique and Genuine.

[20] Kaal H., Must M., Ross E. (2005). Kuiss Vanal Võromaal Eleti. Valimik Korrespondentide Murdetekste.

[21] Reimann N. (2004). Võromaa Kodolugu.

[22] Guerrin T., Karu K. (2014). Võrokõisi Köögi-Ja Söögiraamat.

[23] Luigas I. (2016). Setu Toidud.

[24] Mesikäpp L., Kivisalu I. (2012). Seto Köök: Põlvest Põlve.

[25] Assmuth L., Berglund J., Lundén T., Strandbrink P. (2015). Intertwining identities: The politics of language and nationality in the Estonian-Russian borderlands. Crossings and Crosses: Borders, Educations, and Religions in Northern Europe.

[26] Assmuth L., Bacas J.L., Kavanagh W. (2013). Asymmetries of gender and generation in a post-Soviet borderland. Border Encounters. Asymmetry and Proximity at Europe’s Frontiers.

[27] Kalkun A. (2019). Seto kultuur: Eksootiline võõras ja lähedane oma. Horisont.

[28] Palo A., Külvik M., Palo K., Puura I. (2016). Taimkate. Setomaa.

[29] Kull T., Külvik M., Palo K., Puura I. (2016). Taimestik. Setomaa.

[30] Kukk Ü., Külvik M., Palo K., Puura I. (2016). Taimestik ja selle haruldused. Setomaa.

[31] Jääts I. (2000). Ethnic identity of the Setus and the Estonian–Russian border dispute. Natl. Pap..

[32] Laid E. (1935). Setumaa eestistub kiiresti. Postimees.

[33] (1935). Ringi ümber kodumaa. Postimees.

[34] (1936). Ringi ümber kodumaa. Postimees.

[35] (1937). Ringi ümber kodumaa. Postimees.

[36] Korb A., Kalkun A. (2007). Setod Siberimaal. Sirp.

[37] (1940). Läbi kodumaa linnade. Postimees.

[38] (1936). Setud igatsevad Läti lihapotte. Postimees.

[39] (1936). Ringi ümber kodumaa. Postimees.

[40] (1938). Ringi ümber kodumaa. Postimees.

[41] International Society of Ethnobiology International Society of Ethnobiology Code of Ethics (with 2008 Additions). http://ethnobiology.net/code-of-ethics/.

[42] (2013). The Plant List. www.theplantlist.org/.

[43] Stevens P.F. Angiosperm Phylogeny Website. 2001 onwards. Version 14, July 2017 [and More or Less Continuously Updated Since]. http://www.mobot.org/MOBOT/research/APweb/.

[44] Sõukand R., Kalle R. (2016). Changes in the Use of Wild Food Plants in Estonia: 18th–21st Century.

[45] Mauri M., Elli T., Caviglia G., Uboldi G., Azzi M. RAWGraphs: A Visualisation Platform to Create Open Outputs. Proceedings of the 12th Biannual Conference on Italian SIGCHI Chapter.

[46] Moora A. (1980). Kuidas vanasti marjul ja seenel käidi. Eest. Lood..

[47] Moora A. (1981). Mida vanasti loodusest leivakõrvaseks korjati. Eest. Lood..

[48] Moora A. (1981). Marjad rahvatoidus. Eest. Lood..

[49] Moora A. (1982). Kuidas vanasti kasemahla võeti. Eest. Lood..

[50] Moora A. (1982). Mida vanasti kasemahlast tehti. Eest. Lood..

[51] Moora A. (1984). Kuidas vanasti kadakamarju kasutati. Eest. Lood..

[52] Moora A., Jaagosild I. (1973). Etnograafiamuuseumi kogud rahva toidu uurimise alusena. Etnograafiamuuseumi Aastaraamat.

[53] Bardone E., Pungas-Kohv P. (2015). Changing values of wild berries in Estonian households: Recollections from an ethnographic archive. J. Balt. Stud..

[54] Gustav Vilbaste’s Folklore Collection (1907–1966). Deposited in the Estonian Folklore Archives.

[55] Jakob Hurt’s Folklore Collection (1860–1906). Deposited in the Estonian Folklore Archives.

[56] Estonian Folklore Archiv’s Folklore Collection (1927–1944). Deposited in the Estonian Folklore Archives.

[57] Archive of answers of correspondents of the Estonian National Museum (KV 14; KV 39) (1928–1958).

[58] Vilbaste G. (1993). Eesti Taimenimetused = Nomina Vernacula Plantarum Estoniae.

[59] Taal K., Tomba M., Eichenbaum K. (2010). Suitsuliha Valmistamine Suitsusaunas Vana-Võromaal. http://www.rahvakultuur.ee/vkpnimistu/index.php?page=Public.Knowledge&id=135&PHPSESSID=2cmean2ee96fkgbj4e6ki2u2g2.

[60] Spuhl-Rotalia J. (1897). Kodumaa Marjad.

[61] Pogen O. (1977). Meie Marjad.

[62] Shikov A.N., Tsitsilin A.N., Pozharitskaya O.N., Makarov V.G., Heinrich M. (2017). Traditional and current food use of wild plants listed in the Russian pharmacopoeia. Front. Pharmacol..

[63] Tammeorg J., Kook O., Vilbaste G. (1984). Eesti NSV Ravimtaimed.

[64] Kalle R., Belichenko O., Kuznetsova N., Kolosova V., Prakofjewa J., Stryamets N., Mattalia G., Šarka P., Simanova A., Prūse B. (2020). Gaining momentum: Popularization of Epilobium angustifolium as food and recreational tea on the Eastern edge of Europe. Appetite.

[65] Kalle R., Sõukand R. (2020). The name to remember: Flexibility and contextuality of preliterate ethnobotany from the 1830s, in Pernau, Livonia. J. Ethnoph..

[66] Sõukand R., Kalle R. (2012). The use of teetaimed in Estonia, 1880s–1990s. Appetite.

[67] Tarand A., Jaagus J., Kallis A. (2013). Eesti Kliima Minevikus ja Tänapäeval.

[68] (1933). Ringi ümber kodumaa. Postimees.

[69] (1934). Ringi ümber kodumaa. Postimees.

[70] Remm L., Rünkla M., Lõhmus A. (2018). How Bilberry Pickers Use Estonian Forests: Implications for Sustaining a Non-Timber Value. Balt. For..

